# Apolipoprotein E Epsilon 4 Genotype, Mild Traumatic Brain Injury, and the Development of Chronic Traumatic Encephalopathy

**DOI:** 10.3390/medsci6030078

**Published:** 2018-09-14

**Authors:** Hansen Deng, Angel Ordaz, Pavan S. Upadhyayula, Eva M. Gillis-Buck, Catherine G. Suen, Caroline G. Melhado, Nebil Mohammed, Troy Lam, John K. Yue

**Affiliations:** 1 Department of Neurological Surgery, University of California San Francisco, San Francisco, CA 94122, USA; hansen.deng@ucsf.edu (H.D.); angel.ordaz@ucsf.edu (A.O.); 2 Department of Neurological Surgery, University of California San Diego, San Diego, CA 92093, USA; psupadhy@ucsd.edu; 3 Department of Surgery, University of California San Francisco, San Francisco, CA 94122, USA; eva.gillis-buck@ucsf.edu (E.M.G.-B.); caroline.melhado@ucsf.edu (C.G.M.); 4 Howard Hughes Medical Institute, Chevy Chase, MD 20815, USA; 5 Department of Neurology, University of Utah, Salt Lake City, UT 84112, USA; catherine.suen@hsc.utah.edu; 6 Department of Pathology, University of California San Francisco, San Francisco, CA 94122, USA; nebil.mohammed@ucsf.edu; 7 Department of Oral and Maxillofacial Surgery, University of California San Francisco, San Francisco, CA 94122, USA; troy.lam@ucsf.edu

**Keywords:** apolipoprotein E, chronic traumatic encephalopathy, concussion, genetic risk factors, mild traumatic brain injury, neurodegenerative disorders

## Abstract

The annual incidence of mild traumatic brain injury (MTBI) is 3.8 million in the USA with 10–15% experiencing persistent morbidity beyond one year. Chronic traumatic encephalopathy (CTE), a neurodegenerative disease characterized by accumulation of hyperphosphorylated tau, can occur with repetitive MTBI. Risk factors for CTE are challenging to identify because injury mechanisms of MTBI are heterogeneous, clinical manifestations and management vary, and CTE is a postmortem diagnosis, making prospective studies difficult. There is growing interest in the genetic influence on head trauma and development of CTE. Apolipoprotein epsilon 4 (*APOE-ε4*) associates with many neurologic diseases, and consensus on the ε4 allele as a risk factor is lacking. This review investigates the influence of *APOE-ε4* on MTBI and CTE. A comprehensive PubMed literature search (1966 to 12 June 2018) identified 24 unique reports on the topic (19 MTBI studies: 8 athletic, 5 military, 6 population-based; 5 CTE studies: 4 athletic and military, 1 leucotomy group). *APOE-ε4* genotype is found to associate with outcomes in 4/8 athletic reports, 3/5 military reports, and 5/6 population-based reports following MTBI. Evidence on the association between *APOE-ε4* and CTE from case series is equivocal. Refining modalities to aid CTE diagnosis in larger samples is needed in MTBI.

## 1. Introduction

Mild traumatic brain injury (MTBI) accounts for 70–90% of all traumatic brain injury (TBI), with an estimated 10–15% of MTBI patients reporting persistent cognitive and/or neuropsychiatric deficits at one year post-injury and beyond [1,2,3]. Of particular interest are patients subject to repetitive head trauma in the military or contact sports, contributing to the risk of chronic traumatic encephalopathy (CTE). First coined by New Jersey pathologist Harrison Martland in boxers as “punch-drunk syndrome” and “dementia pugilistica” in subsequent case reports [4,5], this collection of clinical symptoms was termed CTE by British neurologist Critchley in 1949 [6]. In 1973, Corsellis further clarified the diagnosis [7].

**Figure 1:**
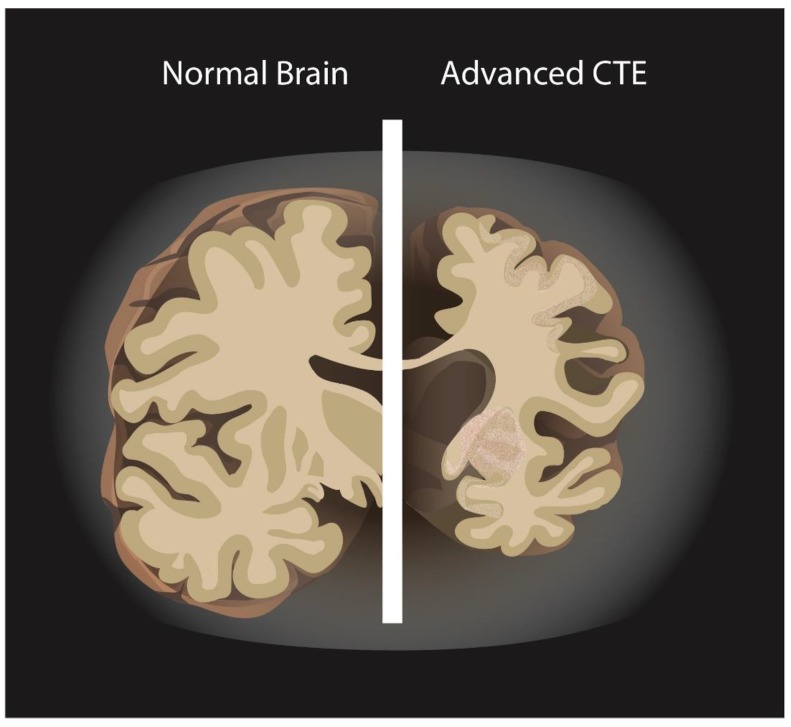
Normal brain vs. Chronic traumatic encephalopathy (CTE).

The National Institute of Neurological Disorders and Stroke (NINDS) defined CTE in 2015 as the abnormal and irregular accumulation of hyperphosphorylated tau (p-tau) in neurons and astroglia around small blood vessels in the cortical sulci [8]. Gross pathology also includes decreased brain weight, enlarged lateral and third ventricles, thinned corpus callosum, fenestrated cavum septum pellucidum and cerebellar tonsillar scarring/loss (Figure 1) [7,9,10]. With repetitive concussions, a progressive spectrum of behavioral, cognitive, and/or motor deficits can manifest 8–10 years [9]. Symptoms include irritability, impulsivity, aggression, depression, short-term memory loss, and suicidal risk; advanced stages are dementia, speech and gait disturbances, and parkinsonism that may overlap with tauopathies such as Alzheimer’s disease and frontotemporal dementia. The diagnosis of CTE through postmortem neuropathology makes understanding of its incidence difficult [11,12,13,14].

There is considerable interest in genetic predispositions that may influence MTBI recovery, since not all patients with repetitive brain trauma will develop CTE [15,16]. The ability to identify individuals early on who are at risk takes precedence. Allelic variants of the apolipoprotein E (*APOE*) gene may be predictors for impaired recovery after MTBI [17,18]. As the predominant lipid transporter in the brain, *APOE* has three molecular isoforms: *APOE epsilon 2* (*ε2*), *APOE epsilon 3* (*ε3*), *APOE epsilon 4* (*ε4*)*.* The six genotypes (*ε2/ε2*, *ε3/ε3*, *ε4/ε4*, *ε2/ε3*, *ε2/ε4*, *ε3/ε4*) vary in their capacity to maintain synaptic function, facilitate neuronal repair, and modulate inflammation following injury [19,20]. Transgenic mice with *APOE-ε4* experience greater excitotoxicity, edema, and ischemia with head trauma [19,21,22]. *APOE-ε4* has been shown to be a strong genetic risk factor for amyloid pathology (e.g., β-amyloid deposition, subsequent neuroinflammation and microglia activation) in Alzheimer’s disease [23,24,25,26]; however its association with tauopathies—the typical pathological hallmark of CTE—is less clear. Recent studies show that *APOE-ε4* may associate with cerebrospinal fluid (CSF) levels of tau and p-tau [23,27,28], as well as possible associations and/or interactions between *APOE* and tauopathies in the setting of β-amyloid deposition.

Evidence in human cohorts has been equivocal, some having reported unfavorable outcome associated with *APOE-ε4* [29,30,31,32,33,34], and others finding no association [35,36,37]. Chamelian et al. observed no association in patients with mild and moderate TBI [36], but in a large cohort of mild, moderate, and severe TBI, Teasdale et al. found worse recovery in children and young adults [37]. Thus, the clinical significance of *APOE-ε4* with respect to neuronal repair and synaptic plasticity deserves further study with specific TBI subpopulations. The challenges of investigating *APOE* genotypes in patients suffering repetitive MTBI and risk developing CTE include the heterogeneity of injury mechanisms, confounding demographics and comorbidities, and the lack of large-scale prospective studies. Towards this goal, we comprehensively review and evaluate the evidence to date on the association of *APOE-ε4* carriers with functional outcomes after MTBI and the diagnosis of CTE.

## 2. Methods

### Study Selection

A comprehensive literature search was conducted using the National Library of Medicine PubMed database for all studies published on the association of *APOE* genotypes with MTBI and/or CTE. Articles published in English through 12 June 2018 were candidates for inclusion using the following search criteria: ((chronic traumatic encephalopathy [title/abstract/MeSH terms] or mild traumatic brain injury [title/abstract/MeSH Terms] or concussion [title/abstract/MeSH Terms]) and apolipoprotein [title/abstract/MeSH Terms]). Two study authors (H.D., A.O.) independently evaluated each article and its accompanying references for scientific merit, with focus on *APOE* allele status in adult patients who experienced MTBI and/or developed CTE, and reached consensus regarding the inclusion of each reference into the current review. Any disagreements were adjudicated independently by the senior author (J.K.Y.). Informed consent was not applicable to this study as it involves no active human subjects.

**Figure 2:**
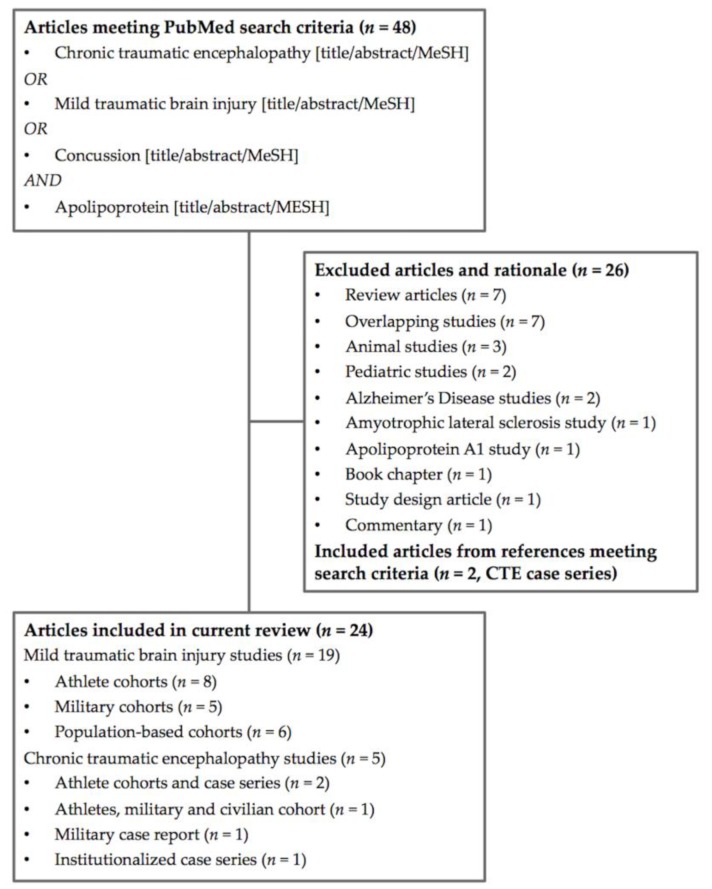
Flowchart of included studies.

Of the 48 unique articles identified, 26 were removed due to the lack of applicability to the current review (seven review papers, seven overlapping studies, three animal studies, two pediatric studies, two studies on Alzheimer’s disease, one on amyotrophic lateral sclerosis, one on apolipoprotein A1, one on study design, one book chapter, and one commentary) (Figure 2). Two articles from the accompanying references on CTE case series, were included [15,38].

A final total of 24 articles, of which 19 studies were on MTBI (eight athletic (four prospective, four retrospective), five military (all retrospective), six population-based (all prospective)) and five studies were on CTE (three sports-related and military case series, one military case report, one institutionalized case series) were deemed fit for inclusion in the current review (Table 1).

## 3. Results

### 3.1. Mild Traumatic Brain Injury in Athletes

Eight studies evaluated associations between *APOE* genotype and MTBI in athletes of various competitive levels (Table 1). While four studies did not note an association between *APOE-ε4* carriers and MTBI outcomes, four others reported otherwise. Kristman et al., 2008, a prospective cohort of 318 various collegiate athletes (25% possessing *APOE-ε4* allele), recorded 28 athletes experiencing concussions, with no association demonstrated between concussion incidence and ε4 [39]. In Tierney et al., 2010, a cross-sectional study of 229 football and female soccer college players (32% with ε4 allele), 48 athletes had prior documented concussion, which did not associate with *APOE-ε4* [40]. Similarly, in 45 retired NFL players (38% having *APOE-ε4* allele) with mean age of 46 years, a 7-year career span, and average number of seven concussions, pathologic neuroimaging findings and neuropsychological impairment did not vary with the genotype [41]. Lastly, a case-control analysis of 128 non-concussed controls and 160 previously concussed rugby players (average of two concussions per player) at all competitive levels did not detect differences in allele frequency between the groups, nor did self-report post-concussive symptoms differ in duration for concussed players [42].

Four investigations found an association with *APOE-ε4* and neuropsychological outcomes following sports-related MTBI. In a prospective cohort of 42 college athletes (36% possessing ε4 allele), Merritt et al., 2016 measured post-concussive symptoms by using the Post-Concussion Symptom Scale (PCSS) within 10 days of MTBI [43]. The PCSS is a 22-item self-reporting measure showing ε4-positive athletes endorsing higher likelihood of physical and cognitive symptoms, with an odds ratio (OR) of 5.25 and 4.75 respectively. A group of 57 collegiate athletes on various teams (35% with ε4 allele) underwent a comprehensive neurocognitive battery across domains of learning, memory, attention, processing speed, executive functioning 14 days postinjury [44]. While mean neurocognitive scores did not differ between allelic groups, ε4-positive athletes exhibited a greater proportion of impaired neurocognitive scores and high-performance variability, which the authors attributed to less efficient and other nonspecific influences of the *APOE-ε4* allele on cognitive processing.

Esopenko et al., 2017, consisting of 38 retired professional hockey players (28% with ε4) and 20 age-matched controls, found that psychiatric complaints in retired athletes were associated with possession of the ε4 allele [45]. In a cross-sectional analysis of 250 collegiate athletes (27% with ε4 allele, 24% with self-reported MTBI history) from various sports, baseline cognitive status were measured by Immediate Post-concussion Assessment and Cognitive Testing (ImPACT) [46]. The authors did not detect differences in concussion susceptibility attributable to *APOE*; however, the baseline reaction times of ε4 carriers were slower compared to other allele groups.

### 3.2. Mild Traumatic Brain Injury in Military Cohorts

Four of the five studies with active-duty personnel or veterans found no clear association between *APOE-ε4*, MTBI susceptibility and/or outcome measures (Table 1). In Dretsch et al., 2017, a retrospective cohort of 458 active-duty soldiers preparing for deployment (mean age 26 years old, 22% with ε4 allele), 36% self-reported prior history of concussion, *APOE* alleles did not associate with having a history of MTBI [47]. In a retrospective study of 160 veterans (mean age 33 years old, 23% with ε4 allele, 53% with military-related lifetime MTBI), Hayes et al., 2017 reported that while MTBI resulted in reduced cortical thickness measured on magnetic resonance imaging (MRI), the *APOE* loci did not interact with MTBI to affect cortical thickness [48].

Han et al., 2009 analyzed whether *APOE* genotype may contribute to change in job status, i.e., reduced duties following mild to moderate TBI in 46 active-duty members (35% with ε4 allele) [49]. Notably, the authors observed that a change in free delay recall on the California Verbal Learning Test-Second Edition (CVLT-II) predicted job status change only in ε4-positive subjects [49]. Pre-deployment data from 120 active-duty soldiers (mean age 27 years old, 29% with ε4 allele, 18% with MTBI history), Emmerich et al. observed that ε4-carriers with MTBI history were associated with increased levels of phospholipids, in particular lysophosphatidylcholine [50]. The *APOE-ε4* protein is less efficient in transporting these lipids, which the authors proposed can exacerbate the effects of injury/disease. Lastly, Nielsen et al., 2018, a cross-sectional study of 47 veterans with MTBI and 40 control veterans, detected lower levels of plasma *APOE* in both groups carrying the ε4 allele [51]. The authors did not observe an association between *APOE* genotype and MTBI status, and found instead that the ε4 allele associated with *APOE* gene promoter methylation and conferred increased susceptibility to combat-related posttraumatic stress disorder (PTSD) [51].

### 3.3. Mild Traumatic Brain Injury in Population—Based Cohorts

In six prospective population-based studies, an association between *APOE-ε4* genotype and MTBI outcome was found in five reports. Liberman et al., 2002 studied a longitudinal series of 72 presenting with mild and 8 with moderate TBI (mean age 41 years old, 23% with ε4 allele) presenting at a trauma center, who were administered neuropsychological testing at 3 and 6 weeks [52]. The first testing showed that ε4-carriers had lower scores on 12 of the 13 tests, of which two were significant- grooved pegboard and paced auditory serial addition task; scores remained lower on 11 of 13 tests by 6 weeks although none were significant, which the authors attributed to slowed recovery with ε4 following TBI [52]. In a prospective matched cohort in Sweden analyzed post-injury symptoms of 31 MTBI subjects (mean age 55 years, 39% with ε4 allele, mean follow-up 20 months) and 62 controls [53]. *APOE-ε4* carriers with MTBI experienced more fatigue than noncarriers with MTBI (58% vs. 32%) and ε4 carrier controls (58% vs. 17%), whereas there was no difference in fatigue level between MTBI ε4 noncarriers and controls.

A prospective study in Norway included 59 MTBI patients (mean age 35 years, 22% with ε4 allele) who received a battery of neuropsychological tests during hospitalization and at 6 months. *APOE* genotype was not found to be a predictor of impairment at either time point; however ε4-positive patients, as with positive computed tomography (CT) findings, associated with reduced improvement in performance at 6 months [54]. In Yue et al., 2017, a prospective cohort of 114 MTBI patients (mean age 43 years, 28% with ε4 allele), verbal memory evaluation using the CVLT-II at 6 months post-injury revealed impaired long-delay free and cued recall for ε4 carriers, along with marginally decreased short-delay cued recall [55]. Yang et al., 2015, a retrospective cohort of 27 individuals with MTBI (mean age 54 years for those without dementia, 60 years with dementia, MTBI history 6 years ago, 30% with ε4 allele) and 10 age-matched controls, found the highest *APOE-ε4* frequency in subjects with MTBI history plus development of dementia (0.250 vs. 0.143 for MTBI without dementia vs. 0.050 for controls) [56]. The authors suggested that *APOE* genotype can be one of many contributing factors to amyloid accumulation.

In 189 patients with MTBI (19% with ε4 allele, mean age 42 years for carriers, 40 years for noncarriers) evaluated at weeks 1 and 6 post-injury, Lee et al., 2017, using the Pittsburgh Sleep Quality Index (PSQI) did not show a difference in recovery of sleep disturbance by *APOE* status [57].

### 3.4. Chronic Traumatic Encephalopathy in Athletes and Veterans

This review examined evidence of *APOE-ε4* genotype associating with CTE development, which is a postmortem pathologic diagnosis limited to case series and reports to date. Evidence on sports-related development of CTE encompassed three case series from McKee et al., Omalu et al., and Stern et al., involving athletes and veterans, the majority of whom were also athletes. In a cohort of 85 subjects with history of repetitive MTBI, 65 cases had confirmed CTE with available *APOE* genotyping (64 athletes and 21 veterans, mean age 60 years old, 29% with ε4 allele, 63% with CTE as sole diagnosis) [15]. The authors did not find the proportion of CTE patients with ε4 allele to be greater than the general population. The brains of 17 subjects (14 former professional athletes in football, wrestling, boxing, and martial arts; three high school football players) were examined after unexpected deaths by Omalu et al., 2011, and the causes of death were as follows: six, five, and three professional athletes died of accidental drug abuse related causes, suicide, and natural causes, respectively; three high school players died from acute accidental trauma while playing football [58]. The mean age was 36 years, and CTE was identified in 11 athletes (10 of 14 professional, 1 of 3 high school) with mean age of 41 years; *APOE* genotyping was available for 12 of 17 athletes and three of these subjects (25%) were determined to possess ε4 allele [58]. Two of 7 CTE-positive professional athletes were ε4-positive (29%), while none of the high school players had any evidence of CTE.

Stern et al., 2013 examined the brains of 36 former athletes with confirmed CTE (29 football players, 3 hockey players, 3 boxers, 1 wrestler; mean age 57 years; leading causes of death are systemic, suicide, overdose, and dementia-related; 35% with ε4 allele) and interviewed the next-of-kin for clinical symptomatology [38]. Ten of 36 subjects also had dementia and stage IV CTE based on severity of p-tau pathology [15]. Although authors did not observe a difference between the *APOE* genotypes of CTE subjects with dementia from those seen in patients with Alzheimer’s disease, notably they found a greater proportion of ε4 homozygotes in CTE patients than expected in a normal, age-matched population [38]. Two clinical subtypes of CTE were reported: one early in the course of the disease manifesting as behavior/mood impairments, and the other exhibiting cognitive impairment later in life. The authors suggested that *APOE-ε4* susceptibility for CTE was largely driven by ε4 homozygotes in the cognitively impaired group [38].

Omalu et al., 2011 published the case report of a 27-year-old Marine Corps Iraqi war veteran, with *APOE* genotype ε3/ε4. The subject experienced repeated mortar blast exposures on active duty prior to honorable discharge and committed suicide by hanging, with subsequent biopsy demonstrating neuropathologic evidence of CTE [14]. Prior to his death, the subject reported persistent mood symptoms and neuropsychological testing revealed impairments diagnosed as PTSD. The authors concluded that the histochemical and immunohistochemical findings of multifocal, neocortical, and subcortical neurofibrillary tangles and neuritic threads were similar to CTE changes previously observed in athletes [14].

### 3.5. Chronic Traumatic Encephalopathy in Leucotomy Patients

Shively et al., 2017 studied post-mortem brain tissues of five institutionalized schizophrenic patients (mean age 78 years at death, 60% with ε4 allele) who received bilateral prefrontal leucotomy, i.e., iatrogenic axonal injury, 4 decades prior to death, which could have contributed to the development of CTE [59]. Results were compared to five age-matched schizophrenic patients (60% with ε4 allele) in the same institution who had not undergone leucotomy. Whereas p-tau and β-amyloid were not notable in the nonleucotomized group, all leucotomy cases had patterns of cortical p-tau distribution in adjacent gray matter that was pathognomonic of CTE lesions in addition to the ε4-carriers having scattered β-amyloid plaque formation. The authors concluded that these findings suggested *APOE* genotype and axonal injury were factors contributing to the development of CTE [59].

## 4. Discussion

There is growing interest over the past 2 decades to improve understanding and prognostication of long-term outcomes after MTBI, with focus on multidimensional risk factors. MTBI commonly occurs collegiate and professional sports, and morbidity is exacerbated by repeated mild trauma inherent in some sports activities [60,61,62]. Continued efforts to improve triage, monitoring and surveillance of patients presenting to the emergency department with concussive symptoms and during their acute follow-up period is increasingly recognized as critical for reducing morbidity. For military personnel (active-duty or retired), there is a high incidence of blast exposure in the battlefield. A possible link between acute MTBI and chronic impairments in subsets of individuals may relate to different *APOE* isoforms and their varying levels of protein function in lipid processing and neuroimmunological activation [63,64,65].

Clinical outcome measures across multiple domains are increasingly used to determine whether *APOE-ε4* carriers are more susceptible to MTBI, fare worse during recovery, and are more prone to developing CTE through repetitive head trauma. Uncovering specific factors contributing to recovery heterogeneity may have profound implications on the patient suffering from concussion. Genetics may in part play a role to influence acute and chronic responses to trauma. In this review, we evaluate the literature on the strength of association of *APOE-ε4* on MTBI and CTE in athletes, military personnel, and the general public, while also discussing the range of clinical measures used by each study.

### 4.1. Role of APOE in the Central Nervous System

The *APOE* protein is a well-known endogenous immunomodulatory agent synthesized in response to injury, and functions to modulate lipid transport, cell membrane and synaptic maintenance, mitochondrial energy production, neuronal repair, and synaptogenesis [63]. The *APOE* gene is located on chromosome 19 and is highly polymorphic, resulting in a range of genotypes. *APOE* ε2 and ε3 have two and one cysteine residues, respectively, capable of detoxifying cytotoxic products of lipid peroxidation, whereas ε4 has two arginine residues that lack such ability. Differences in the tertiary structure and charge distribution of the *APOE-ε4* isoform impair its capability to orchestrate these functions. Evidence in the literature attributes *APOE-ε4* with a proinflammatory state and dysregulation of cerebral perfusion [19,20,65,66,67], along with changes in the blood-brain-barrier and cerebral edema in mice models [68]. While the proinflammatory influence can confer an evolutionary survival advantage in populations with high-risk exposure to infectious diseases [64], it is detrimental in a variety of neurological disorders [63]. As a regulator of neuronal repair for deep cerebral structures including the hippocampus, entorhinal complex, parahippocampal gyrus, and basal ganglia, *APOE* modulates regions that are crucial to memory consolidation and sensitive to damage from blunt head trauma [69,70].

The best evidence linking *APOE-ε4* with poor outcomes comes from studies of severe TBI, with patients experiencing worse cognitive and functional impairments, β-amyloid deposition, prolonged coma, as well as a synergistic influence on the risk of developing Alzheimer’s disease [33,71,72,73]. Understanding the role of *APOE-ε4* in MTBI has been challenging, as the set of outcome measures to encompass the range of functional, mood, cognitive, and behavioral domains is expansive while focal deficits may be specific to the individual. However, progress has been made regarding the characterization of specific risk factors after MTBI. Likewise, chronic mild head trauma causing dementia pugilistica was first described 1928 in boxers and now studied in an increasing number of sports and military personnel [5]. Chronic traumatic encephalopathy can present after a latent period as a composite syndrome of mood, neuropsychiatric, and cognitive abnormalities [58]. The histomorphologic features of CTE in a football player were first described in Omalu et al., 2006 [74], which the authors further defined in 2011 with additional cases as a distinct cerebral tauopathy of neurofibrillary tangles (NFTs) and neuritic threads (NTs), with or without the presence of amyloid plaques [58]. In contrast to 28% of the population that possesses at least one *APOE-ε4* at baseline [18], an estimated 57% of CTE individuals are carriers of the ε4 allele [75]. The potential for genetic predisposition is not entirely clear and challenging to study due to the need for neuropathologic diagnosis on autopsy, which limits sample size.

### 4.2. Evidence on APOE and Mild Traumatic Brain Injury

Current research on the association between *APOE-ε4* and MTBI can be better understood through classifying the outcome measures of interest into four categories and evaluating the strength of findings according to each measure: (i) susceptibility for MTBI based on self-reported history of documented concussion, (ii) neuroimaging implications, (iii) acute to subacute/chronic functional disability, and (iv) test battery for neurocognitive and neuropsychological impairment. 

Whether *APOE-ε4* confers a genetic predisposition to TBI susceptibility has important ramifications on current standards of primary prevention, particularly for athletes and military personnel who possess the isoform. Four large athletic cohorts spanning several sports and competitive levels, and one study in active-duty soldiers examined self-reported history of concussions identified by sport-medicine professionals [39,40,42,46,47]. These studies consisted primarily of young adult males, for which *APOE* genotype did not impart increased risk of sustaining a MTBI. The proportion of ε4-carriers in athletes and soldiers ranged from 22–32%, similar to the general population. While the ε4 allele itself did not influence susceptibility, there was a 3-fold risk for subsequent concussions in subjects with prior concussions [39,76]. Future investigations on the possibility of polygenetic risk factors for MTBI will be of interest [40,51].

Intracranial damage from MTBI can be occult using current clinical standards of neuroimaging such as CT and conventional MRI. It is increasingly recognized that while CT-positive pathology indicates worse prognosis, CT-negative imaging is not necessarily a sensitive measure for the absence of intracranial injury. In fact, up to 30% of MTBI patients who are CT-negative can be MRI-positive for intracranial injury, specifically axonal shear and gliding contusions [77]. Yue et al., 2017 found that 25% of MTBI patients had intracranial pathology on initial head CT scan within 24 h of injury, and *APOE-ε4* did not accurately predict the presence of acute CT-positive lesions [55]. Casson et al., 2014 conducted extensive MRI studies with susceptibility weighted imaging (SWI) and diffusion tensor imaging (DTI) in a cohort of retired professional football players experiencing a mean total of nineconcussions over their lifespan, 38% of whom were ε4 carriers [41]. While most players (87%) did not have MRI findings of brain injury, 13% had reduced fractional anisotropy values indicative of chronic injury, which correlated with *APOE-ε4* carrier status. While *APOE-ε4* may not predispose patients to radiographic evidence of TBI after a single injury, axonal injury is a progressive process rather than from a single event [78]. Hence the possibility of *APOE-ε4* to confer susceptibility to white matter injury with repetitive MTBI deserves intensive prospective study.

Prior landmark studies by Teasdale et al. in 2005 and Ariza et al. in 2006 examined TBI patients across all injury severities and found *APOE-ε4* carriers to have worse symptoms and functional recovery [37,79]. However, our review finds this genetic relationship less clear for MTBI. On one hand, ε4-positive college athletes were more likely to endorse physical and cognitive complaints at 1 week postconcussion [43] and fatigue on long-term follow-up [34]. However, in a large cohort of rugby players, Abrahams et al. did not find a difference in the duration of post-concussive symptoms, possibly in part due to the binary outcome measure of more or less than 1 week of symptoms [42]. It is known that MTBI recovery remains variable within the first few weeks, where intervention, rehabilitation, medical and/or behavioral management may impart greater effects than if instituted months postinjury. Secondary injury and evolving intracranial processes can impact brain physiology and recovery across acute and subacute recovery, where *APOE* may continue to modulate immune status and neuroinflammation [20,63,64]. On the other hand, a study on sleep disturbance did not find worse outcomes associated with *APOE-ε4* [57], and Casson et al., 2014 reported a higher prevalence of depression in retired football players (33% vs. 15–20% in the general population) independent of *APOE* status [41].

There is an overall movement towards consensus measures across cognitive domains of learning and memory, attention, processing speed, executive function, and others after MTBI [80,81,82]. Results to date among studies have been mixed, both in general and in the context of *APOE*. Cognitive scores in concussed collegiate athletes (using the Brief Visuospatial Memory Test-Revised) and former athletes (using CogState battery and Cambridge Brain Science) did not differ in any subdomains by *APOE* genotype, except for ε4-athletes having greater score variability [44] and ε4-alumni self-reporting more psychiatric complaints [45]. When the CVLT-II was used; however, *APOE-ε4* carriers suffering MTBI presenting to Level I trauma centers [55] and in the military [49] exhibited worse long-delay verbal memory compared to patients without ε4. Other notable differences include manipulative dexterity in motor function and auditory processing and flexibility at 3 weeks post-injury [52], and a global index incorporating these measures predicted less improvement in ε4 carriers at 6 months [54]. Thus, in contrast to the moderate to severe TBI literature, these studies highlight that after MTBI, specific subdomains of neurobehavioral outcomes, particularly those of memory consolidation, retrieval and processing, may be modulated and/or impacted by *APOE-ε4* status.

### 4.3. Evidence on APOE and Chronic Traumatic Encephalopathy 

Given recent developments in the diagnosis and staging of CTE these past two decades, our review shows that the relationship between *APOE* and CTE is in need for further study [15]. Of 112 total former athletes and veterans confirmed with CTE to date (with the majority being football players) who received *APOE* genotyping, 30% were ε4 carriers [15,38,58]. Systemic illness, suicide, and drug overdose were the most common causes of death of CTE patients. Along with mood lability and disinhibition, patients begin to exhibit progressive worsening of short-term memory loss, executive dysfunction, and loss of attention and concentration 8–10 years after experiencing repetitive MTBI [9]. Similar to impaired memory processing ε4-carriers suffering MTBI, ε4-individuals-particularly homozygotes-with CTE suffered worse cognitive difficulties (episodic memory, executive function, attention and concentration) prior to death, based on the medical records and blinded interviews conducted with next-of-kin [38]. 

In theory, because of neurotoxic effects on the mitochondria and cytoskeleton conferred by the dysfunctional *APOE-ε4* isoform, elevated risks are present for neurodegeneration through widespread deposition of p-tau following repeated injury [83,84]. In the context of limited patient samples, however, no studies thus far have identified increased susceptibility for developing CTE with the ε4 allele. Findings of CTE rely heavily on former athletes with long careers, and a majority of the veterans with CTE had MTBI exposure through sports. The literature reveals that mean age at death ranged from 41 to 60 years, football was the most common sport, and the average career time was 12 to 15 years. Stages I to IV of CTE correlate with the severity of hyperphosphorylated tau pathology, although with multifocal axonal disruption and loss in deep cortex and white matter regardless of the CTE stage [9]. In the largest study to date [15], mean age and new symptomology for each progressive stage were as follows: 28 years old, headache and loss of attention/concentration; 44 years old, depression/lability and short-term memory loss; 56 years old, cognitive impairment, executive dysfunction, visuospatial abnormality; 77 years old, dementia and profound short-term memory loss.

Research in CTE, a progressive tauopathy with distinctive histologic findings and nonspecific clinical symptoms, through autopsy-based case series is limited by the ascertainment bias of using reports from the next-of-kin of the deceased. Additionally, current literature lacks matched controls with exposure of repetitive MTBI but did not develop CTE. Evidence to date does not have the strength to suggest *APOE* status as a predictor of developing CTE; however, clinical criteria for diagnosing CTE need to be further established to facilitate prospective cohort studies for validation.

### 4.4. Limitations and Future Directions

The goal of this investigation was to explore the possibility of *APOE-ε4* influence on MTBI and CTE, and studies on other genes which may play similar roles were not included for analysis. Given the relatively small number of studies measuring a variety of outcome types in each patient cohort, no formal grading of the level of evidence or bias was performed, limiting this study to an overview of the diverse approaches underway in understanding the phenotypic manifestation of a possible genetic risk factor in the context of both MTBI and CTE. While the difference in verbal memory attributed to *APOE-ε4* is statistically significant, its deleterious impact on recovery and generalizability to the populations at large remain uncertain, in part due to English language limitations inherent to the CVLT-II. 

Several hypotheses have been proposed regarding the role of *APOE-ε4* in neurodegeneration following head trauma, including but not limited to the deposition and clearance of Aβ peptides and formation of plaques, dysregulation of neuronal signaling, and abnormal phosphorylation of tau to form neurofibrillary tangles. However, the molecular mechanisms of these ε4-mediated detrimental effects remain largely unknown. Presently, CTE is a diagnosis that can only be made with neuropathological analysis from wide sampling of the brain. Whether there is a primary mechanism, such as overactivated extracellular signal-regulated kinase (ERK) or tau phosphorylation causing neuronal death (leading to CTE over time) [85], cannot be extrapolated using post-mortem brain tissue analysis. 

The studies examined in the current review are also limited by age. In the athletic cohorts with MTBI, most studies (6 of 8) examined subjects with a mean age of 18–21 years, of which three studies showed decreases in neurocognitive tests in ε4-carriers. Of the five military studies, mean age was generally 20–30 years. The six population-based cohorts with MTBI consisted of an older population with mean age 35 to 55 years, of which ε4-carriers showed associations with decreased neurocognitive test performance, more subjective fatigue, and dementia. As CTE is a post-mortem diagnosis, most cases to date are in the older age range (mean age in 50s). Undoubtedly, age can be a modifier and a confounder of *APOE-ε4* effects, and studies have shown age-dependent alterations in CSF beta-amyloid markers for *APOE-ε4* carriers vs. noncarriers [86,87], as well as *APOE* genotype prevalence and associated risk [25,88]. Future studies targeting *APOE* associations with MTBI outcomes and CTE should accordingly evaluate and control for age.

The prevalence of age-related comorbidities in subjects with more severe stages of CTE, along with the presence of other neurological disorders such as motor neuron disease, Alzheimer’s disease, Lewy body disease, and frontotemporal degeneration, limit the generalizability of the studies included in this review. Furthermore, to aid understanding of MTBI exposure leading to CTE, additional history of the TBIs themselves are is needed, e.g., characteristics of each incident, frequency, and the time between subsequent concussions to allow for recovery. To date, of the 5 military studies with MTBI evaluated in the current review, the influence of *APOE-ε4* status on outcome after MTBI remains unclear: one study of 458 active-duty subjects showed a trend of increased prior concussions in ε4-carriers, one study of 87 veterans showed interaction between ε4, PTSD and DNA methylation, one study of 120 veterans showed interactions between ε4 status and diagnosis of TBI and PTSD, one study of 53 active-duty subjects showed a relationship between job status and memory performance associated with ε4-carriers, and one study on cortical thickness was negative. Outcomes after military TBI are undoubtedly multifactorial, and future large genetic studies are necessary to delineate whether *APOE* and other genetic risk factors exist for the military setting. The effects of sports-related, military, and civilian-setting concussions need to be compared in detail to characterize the heterogeneous nature of MTBI. Future prospective investigations will be necessary not only to elucidate the primary mechanisms of *APOE* in mild head trauma but to understand its relationship with findings in neuroimaging, epigenetics, and biomarkers.

## 5. Conclusions

In general, presence of the *APOE-ε4* allele does not increase the susceptibility for MTBI. Post-injury, the ε4 isoform of this immunomodulatory gene is associated with impaired cognition, most pronounced in the subdomains of memory consolidation, retrieval and processing. This may be in part due to decreased capacity by the ε4 protein to modulate neuronal repair in deep brain structures responsible for memory processing. *APOE-ε4* carriers, with repetitive injury, are more likely to sustain white matter injury detectable on neuroimaging in the form of axonal damage. CTE is a progressive neurodegenerative process that manifests approximately a decade after repeated exposure to MTBI and/or concussion. While the proportion of CTE subjects with *APOE-ε4* is no greater than the general population, ε4 carriers may exhibit elevated cognitive difficulties during the progression of their disease course. Future prospective studies are needed to validate *APOE-ε4* status as a risk factor for impairment after MTBI, and to establish premortem clinical criteria of diagnosing CTE to better understand the role that *APOE-ε4* may have early in the disease.

## Figures and Tables

**Table 1:** Summary of included studies and findings for *APOE* allelic variants.

Search Criteria [Title/Abstract/MeSH Terms]: (Chronic Traumatic Encephalopathy or Mild Traumatic Brain Injury or Concussion) and Apolipoprotein E						
**MTBI in Athlete Cohorts (8 Studies)**						
**Author and Year**	**Study Type**	***N***	**Sex; Age**	**Description**	**Outcome Measures**	**Results**
Cochrane et al., 2018	MTBI, prospective, athletes	250 collegiate athletes (95 football, 58 baseball/softball, 67 soccer, 18 basketball, 2 cross-country/track and field)	184 male, 66 female; 19.0 ± 1.3 years old	*APOE*-ε2 carriers (*n* = 28), *APOE*-ε3 carriers (*n* = 128), *APOE-ε4* carriers (*n* = 68), 24% self-reported a history of prior concussion.	Self-reported history of concussions and neurocognitive performance from Immediate Post-concussion Assessment and Cognitive Testing (ImPACT).	Apo-ε4 carriers had significantly slower reaction time (*p* = 0.002) as measured by the ImPACT.
Kristman et al., 2008	MTBI, prospective, athletes	318 collegiate athletes (43 football, 26 field hockey, 32 basketball, 23 ice hockey, 45 lacrosse, 49 rugby, 53 soccer, 47 volleyball)	164 male, 154 female, mean age 20.5 ± 2.4 years (range 17–31)	79 (24.8%) *APOE-ε4*(+) athletes	Concussions in athletes were diagnosed by the sport-medicine team and verified by a sport-medicine physician.	In the proportional hazards model, presence of the *APOE-ε4* was not significantly associated with concussion (*p* = 0.68).
Merritt et al., 2016	MTBI, prospective, athletes	42 collegiate athletes (sports not defined)	35 male, 7 female; subjects with mild concussions (14.3% LOC); ε4 carriers aged 19.9 ± 1.4 years, noncarriers aged 20.0 ± 1.6 years	15 (35.7%) *APOE-ε4*(+) athletes, and 27 (64.3%) *APOE-ε4*(−) athletes	Team physicians determined TBI and concussed athletes were referred for neuropsychological testing post-injury. The Post-Concussion Symptom Scale was used to evaluate self-report symptoms. Physical and cognitive symptom clusters were each dichotomized into “symptoms present” versus “symptoms absent” groups	ε4-carriers associated with “symptoms present” group; ε4(+) athletes more likely to endorse physical symptoms than ε4(−) athletes (odds ratio (OR) = 5.25 (1.33–20.76); *p* = 0.015)). For cognitive symptoms, ε4-carriers associated with “symptoms present” group; ε4(+) athletes more likely to endorse cognitive symptoms than ε4(−) athletes, (OR = 4.75 (1.23–18.41), *p* = 0.020)
Merritt et al., 2018	MTBI, prospective, athletes	57 collegiate athletes (16 football, 11 basketball, 8 lacrosse, 7 rugby, 7 hockey, 4 soccer, 2 wresting, 2 other).	44 males, 13 females; ε4 carriers 20.3 ± 1.4 years old, ε4 noncarriers 20.3 ± 1.4 years old	20 athletes (35.1%) *APOE-ε4*(+); 37 athletes (64.9%) *APOE-ε4*(−)	Neurocognitive test battery including learning and memory, attention, processing speed, and executive functions: Brief Visuospatial Memory Test, Hopkins Verbal Learning Test; Symbol-Digit Modalities Test; Vigil Continuous Performance Test; Digit Span Test from the WAIS-III; Trail-Making Test; Penn State University Cancellation Test; Stroop Color-Word Test; ImPACT.	No significant differences were seen between athletes with and without the *APOE-ε4* allele when examining mean neurocognitive scores (all *p* > 0.05). However, ε4(+) subjects were more likely to show a greater number of impaired neurocognitive scores post-injury compared to ε4(−) athletes (*p* < 0.05). More ε4+ athletes (40.0%; 8 of 20) fell in the impaired group compared with ε4- athletes (16.2%; 6 of 37; *p* = 0.046). *APOE-ε4*(+) athletes demonstrated greater neurocognitive variability than athletes without *APOE-ε4* (*p* < 0.05).
Abrahams et al., 2017	MTBI retrospective, athletes (rugby)	288 rugby athletes (121 high school, 116 club, 51 professional)	Males; controls younger than concussed group (19.2 ± 3.5 years vs. 20.5 ± 4.4 years; *p* = 0.008)	The concussed group (*N* = 160) reported 1.9 ± 1.0 prior concussions (45%: 1; 34.4%: 2; 8.8%: 3; 11.9%: 4 concussions)	Self-reported duration of symptoms (<1 week versus ≥1 week)	*APOE-ε4* isoforms did not differ significantly between concussion and control groups. *APOE-ε4* isoform frequency distribution did not differ significantly between duration of symptoms groups
Casson et al., 2014	Chronic brain damage, retrospective cross-sectional, athletes	45 retired National Football League (NFL) players	45 males; 45.6 ± 8.9 years old (range, 30–60 years)	Athletes reported 6.9 ± 6.2 concussions (maximum 25) throughout their careers	MMSE, dysarthria, pyramidal system dysfunction, extrapyramidal system dysfunction, cerebellar dysfunction, depression, PHQ9, ImPACT. susceptibility weighted imaging (SWI) and diffusion tensor imaging (DTI) evaluation for brain injuries	*APOE-ε4* allele present in 38% of players, a larger number than expected in the general male population (23–26%). No association of the *APOE-ε4* genotype with anatomical magnetic resonance imaging (MRI), clinical neurologic, depression, or neuropsychological test results
Esopenko et al., 2017	MTBI, retrospective, alumni athletes	33 retired National Hockey League (NHL) players	53 males; alumni athletes 54.3 ± 10.4 years old, controls 53.4 ± 10.2 years old,	Concussions in athletes were 4.8 ± 2.7; in controls were 0.6 ± 0.8; 9 alumni and 4 controls were ε4(+); 1 ε4/ε4, 2 ε2/ε4, rest were ε2/ε3 or ε3/ε3	Self-reported history of concussion, cognitive function and questionnaires on psychosocial/psychiatric function	ε4-carriers associated with increased psychiatric complaints (*p* = 0.009) but not objective cognitive performance. Executive function associated with number of concussions after accounting for confounders (β = −0.55 and −0.39, *p* = 0.005 and *p* = 0.039 for concussion and age, respectively)
Tierney et al., 2010	MTBI, retrospective, athletes	196 collegiate athletes (163 football; 33 soccer)	163 male football and 33 female soccer athletes, age was 19.7 ± 1.5 years old	48 (24.5%) with 1 concussion, 9/48 (4.6%) with >1 concussions. ε2 and ε4 present in 35 (17.7%) and 62 (31.9%) athletes.	Self-reported history of concussions.	No association between ε4 and ε2 carriers and history of concussion. Significant association (OR = 9.8; *p* = 0.05) between carrying *APOE* rare alleles and prior concussions.
**MTBI in Military Cohorts (5 Studies)**						
**Author**	**Study Type**	***N***	**Sex; Age**	**Description**	**Outcome Measures**	**Results**
Dretsch et al., 2017	MTBI, retrospective, active-duty military	458 military members from two brigade combat teams preparing to deploy to Iraq and Afghanistan	430 male, 28 female; age was 26.0 ± 7.0 years	History of concussion in 36.5%, with 10.7% having 3+ concussions. 38% (162/430) of men with 1+ concussions, vs. 18% (5/28) of women	Self-reported history of concussions	*APOE* not associated with risk for concussions. Of ε3/ε4 or ε4/ε4 soldiers, 44.1% (41/93) had a history of one or more prior concussions compared with 34.5% (126/365) of the comparison group (OR = 1.50; *p* = 0.087)
Emmerich et al., 2016	MTBI, retrospective, active-duty military	120 demographically matched soldiers with prior deployment to Iraq or Afghanistan (21 with TBI, 34 with posttraumatic stress disorder (PTSD); 13 with TBI + PTSD; 52 controls)	120 males; TBI subjects’ age was 25.9 ± 1.4 years, PTSD subjects’ age was 26.4 ± 1.3 years, TBI + PTSD subjects’ age was 29.9 ± 1.6 years old; control subjects’ age was 26.7 ± 1.0 years	85 *APOE-ε4*(−) (control *n* = 37, TBI *n* = 16, PTSD *n* = 24, TBI + PTSD *n* = 8); *APOE-ε4*(+) *n* = 35 (control *n* = 15, TBI = 5, PTSD = 10, TBI + PTSD *n* = 5)	Self-reported history of TBI, including military-associated injury and all other traumas; alcohol use; medical history; prior deployments; current medications; PTSD symptoms; and depression. Blood samples for lipidomic analysis.	ε4(+) subjects exhibited higher plasma phospholipids levels than their ε4(−) counterparts within diagnostic groups. ε4 noncarriers showed decreased saturated fatty acids (SFA)- and monounsaturated fatty acids (MUFA)-containing phosphatidylethanolamine (PE) species for TBI, PTSD, and TBI+PTSD groups, compared with controls. ε4 carriers showed no significant differences between TBI and PTSD groups for SFA- and MUFA. Interaction between ε4-carriers and diagnosis of TBI+PTSD on MUFA-containing lysophosphatidylcholine (LPC) and lysophosphatidylethanolamide (LPE) species.
Han et al., 2009	Mild to moderate TBI, retrospective, active-duty military	53 military personnel	42 male, 4 female; Mean age of *APOE-ε4*(+) was 22.6 ± 3.8 years, mean age of *APOE-ε4*(−) was 25.2 ± 6.1 years	16 *APOE-ε4*(+), 30 *APOE-ε4*(−)	Job change (reduction in duties for any reason e.g., medical hold, rehabilitation or assignment to light/limited duties (*n* = 24), reduction due to TBI (*n* = 3), referral to Medical Board (*n* = 3), or administrative separation (*n* = 1)), using neuropsychological assessments	In ε4-carriers, job status was determined by a long-delay free recall on the CVLT-II. If the percent change between long-delay free recall and short-delay free recall (defined as ((long-delay free recall raw score) minus (short-delay free recall raw score))/(short-delay free recall raw score)) >3.55%, subjects correctly predicted as no change in work status (85.7% accuracy). If the percentage change was <3.55%, subjects were correctly predicted to have a change in their job status with 88.9% accuracy
Hayes et al., 2017	MTBI, retrospective, veterans	160 veterans of OEF/OIF and/or New Dawn	149 male, 11 female; non-MTBI aged 32.9 ± 8.9 years, MTBI aged 30.6 ± 8.1 years	55 with no MTBI, 105 with MTBI. ε4(+): 10/55 with no MTBI, 27/105 with MTBI	Linear models examined the main effect of *APOE* (ε4-carriers, *n* = 37; non-ε4 carriers, *n* = 123) and MTBI × *APOE* interaction factor on cortical thickness.	No main effect of *APOE* on cortical thickness; no MTBI/*APOE* interaction.
Nielsen et al., 2018	MTBI, retrospective, veterans	87 veterans with or without MTBI	Demographics not published	47 veterans with MTBI and 40 controls	Hierarchical linear regression to evaluate the association between DNA methylation, MTBI, and *APOE* genotype with plasma *APOE*, controlling for age, sex, population structure, depression and PTSD	Plasma *APOE* associated with PTSD severity (*p* = 0.013). Higher *APOE* levels in ε3/ε3 compared to ε4 carriers (*p* = 0.031). Plasma *APOE* was associated with DNA methylation at CpG sites −877 (*p* = 0.021), and −775 (*p* = 0.014). The interaction factor ε4 × PTSD was associated with DNA methylation at CpG −675 (*p* = 0.009)
**MTBI in Population-Based Cohorts (6 Studies)**						
**Author**	**Study Type**	***N***	**Sex; Age**	**Description**	**Outcome Measures**	**Results**
Lee et al., 2017	MTBI, prospective, population-based	189 patients from emergency departments (ED) of three hospitals	76 male, 113 female; Mean age of ε4(+) was 42.2 ± 14.7 years and ε4(−) was 40.1 ± 15.2 years	35 ε4(+), 154 ε4(−)	1st week post-mTBI and 6th week post-mTBI) sleep assessments, using the Pittsburgh Sleep Quality Index (PSQI)	No difference in PSQI at baseline and week 6 between ε4-carriers and noncarriers. Both ε4 carriers and noncarriers exhibited improvement in overall PSQI scores between baseline and week 6 follow-up (carrier: baseline 8.3 ± 3.9, 6th week: 7.4 ± 4.9, *p* = 0.05; noncarrier: baseline 8.5 ± 4.4, 6th week: 8.1 ± 3.8, *p* = 0.03)
Liberman et al., 2002	MTBI, prospective, population-based	87 adult patients presenting with mild or moderate TBI to a shock trauma center	48 males, 32 females; <30 years of age (*n* = 25), 30–49 years old (*n* = 28) and ≥50 years old (*n* = 27)	18 ε4(+), 62 ε4(−)	13 neuropsychological tests administered twice at 3 and 6 weeks post-injury	90% with MTBI; 18 (22.5%) ε4-carriers, who had lower scores on 12 of 13 neuropsychological outcomes at visit 1 compared to noncarriers, 2 were significant (grooved pegboard test, *p* = 0.005; paced auditory serial addition task 2.8-s trial, *p* = 0.004). At visit 2, ε4(+) had lower adjusted mean scores on 11/13 neuropsychological outcomes, though none were statistically significant.
Muller et al., 2009	MTBI, prospective, population-based	59 patients with MTBI	47 male, 12 female; Mean age 35.1 years (range 18–74)	13 *APOE-ε4*(+), 46 *APOE-ε4*(−)	GCS in ED, head computed tomography (CT) and MRI, neurophysiological assessments at baseline and 6-months. Serum S100B was measured.	GCS < 15, TBI on CT/MRI, and serum S-100B > 0.14 μg/L predicted impaired cognitive performance at baseline and 6-months while *APOE* did not. *APOE-ε4* genotype was the only independent factor significantly predicting less improvement from baseline to 6-months.
Sundstrom et al., 2007	MTBI, prospective, population-based	31 MTBI patients and 62 matched controls	18 male, 13 female; Mean age 55.2 ± 13.6 years	*APOE-ε4* present in 38.7% of MTBI subjects and controls	Self-reported pre and postinjury fatigue, anxiety, depression and sleep disturbance was compared within-group and between groups	In MTBI, fatigue was more commonly reported among ε4 carriers (58%) than noncarriers (32%). MTBI ε4-carriers were more often fatigued than controls with ε4 (58% vs. 17%, *p* = 0.02). No significant between-group differences between MTBI and controls without ε4
Yang et al., 2015	MTBI, prospective, population-based	21 MTBI patients without dementia, 6 MTBI patients with dementia, and 10 controls without MTBI	15 male, 22 female; (controls: 2 M, 8 F, aged 50.6 ± 6.8 years; MTBI without dementia: 9 M, 12 F, aged 53.7 ± 7.9 years; MTBI with dementia: 4 M, 2 F; aged 60.0 ± 7.5 years)	ε4 carriers: 5 of 21 MTBI without dementia, 4 of 6 MTBI with dementia, 1 of 10 controls	MMSE, amyloid-PET	ε4 frequency high in MTBI patients with dementia (*p* = 0.049). Linear regression between *APOE-ε4* and average amyloid standardized uptake value ratio (SUVR) showed significant correlation for all subjects (*p* < 0.05)
Yue et al., 2017	MTBI, prospective, population-based	114 MTBI patients	76 male, 38 female; aged 49.6 ± 13.6 for ε4(+); aged 39.7 ± 16.5 years for noncarriers	79 ε4(−), 35 ε4(+)	6-month verbal memory using the CVLT-II, including Short-Delay Free Recall (SDFR), Short-Delayed Cued Recall (SDCR), Long-Delay Free Recall (LDFR), and Long-Delay Cued Recall (LDCR).	ε4-carriers associated with long-delay verbal memory deficits (LDFR: B = −1.17 points, 95% CI (−2.33, −0.01), *p* = 0.049; LDCR: B = −1.58 (−2.63, −0.52), *p* = 0.004), and a marginal decrease on SDCR (B = −1.02 (−2.05, 0.00), *p* = 0.050). CT pathology was the strongest predictor of decreased verbal memory.
**CTE in Athlete and Military Cohorts (4 Studies)**						
**Author**	**Study Type**	***N***	**Sex; Age**	**Description**	**Outcome Measures**	**Results**
Stern et al., 2013	CTE, retrospective, athletes	36 athletes (29 football (22 pro, 4 college, 3 high school), 3 hockey, 1 wrestling, 3 boxing (1 pro, 2 amateur)) with neuropathologically confirmed CTE	All male; aged 56.8 ± 21.9 years (range 17–98)	*APOE-ε4* genotype distribution was 3% ε2/ε3, 63% ε3/ε3, 26% ε3/ε4, and 9% ε4/ε4	Next-of-kin interviewed for neuropsychiatric, social/occupational histories, dementia, depression, changes in cognition, behavior, mood, motor function, and ADLs	Proportions of *APOE* genotypes (i.e., ε4/ε4, ε4 carriers, and ε4 noncarriers) in this CTE sample were significantly different from those found in an age-matched normative sample (*p* < 0.05). Primary difference between this CTE sample and population norms was a greater proportion of ε4/ε4 in this sample (*p* < 0.05). More ε4/ε4 in the cognition group than expected (*p* < 0.05). Relative proportions of *APOE* in the 10 subjects with dementia did not differ significantly from those seen in Alzheimer’s (*p* > 0.05)
Mckee et al., 2013	CTE, retrospective, athletes, military veterans, and civilians	80 athletes (22 veterans), 3 military veterans, 1 civilian with history of falls, 1 civilian with history of self-injurious head banging behavior; 18 age- and sex-matched controls	84 males, 1 female; Mean age of 54.2 ± 23.3 years (age range 14–98)	Of all subjects, including controls, 21 were carriers of Apo-ε4, 5 were homozygous for Apo-ε4	Post-mortem brains of subjects with histories of repetitive MTBI were analyzed for evidence of CTE. Hyperphosphorylated tau pathology ranged in severity from focal pathology in the frontal lobe to a more global tauopathy, allowing for a progressive staging of pathology in these subjects. *APOE* genotyping was performed	In the 68 subjects diagnosed with CTE, the proportion of carrying at least one *APOE-ε4* allele was not significantly different than that observed in the general population (*p* = 0.334)
Omalu et al., 2011	CTE, case series, athlete	14 pro athletes (8 football, 4 wrestling, 1 boxing, 1 mixed martial arts, 3 high school football)	Subjects male; age range 16–52 years	ε3/ε3 in 6 athletes (60%), ε3/ε4 in 2 athletes (20%), ε2/ε3 in 1 athlete (10%), ε2/ε4 (10%) in 1 athlete. 9 of the pro athletes (90%) with at least 1 ε3 allele. 7 of 10 pro athletes with known *APOE* genotype had CTE (70%). For the 3 deceased high school football players, the *APOE* genotype in 1 case could not be determined (blood samples were not available), and the genotypes in the other 2 were ε3/ε3 and ε3/ε3	Histochemical and immunohistochemical brain tissue analysis for CTE changes and apolipoprotein E genotyping	Three pro athletes carried *APOE-ε4*, two of which (ε3/ε4 genotype) were positive for CTE, and the remaining (ε2/ε4 genotype) negative for CTE
Omalu et al., 2011	CTE, case report, military	Case report of a military individual	27-year-old male	The *APOE* genotype was ε3/ε4	Histochemical and immunohistochemical brain tissue analysis for CTE changes	Autopsy, as well as gross and histomorphological examination of this brain revealed CTE changes similar to those observed in USA athletes
**CTE in an Institutionalized Cohort (1 Study)**						
**Author**	**Study Type**	***N***	**Sex; Age**	**Description**	**Outcome Measures**	**Results**
Shively et al., 2017	Leucotomy, case series, institutionalized	5 institutionalized patients with schizophrenia and history of surgical leucotomy, with post-diagnosis survival of >40 years	2 male, 3 female; Ages were 67, 70, 77, 87, and 89 years	Three of 5 are *APOE-ε4* carriers, with the other 2 having ε3/ε3 genotype	Immunohistochemistry for abnormally hyperphosphorylated tau, beta-amyloid, antigen CD68; H&E stains on tissue sections for general morphology/structure	The three ε4-carriers showed scattered β-amyloid plaques in the overlying gray matter, which were not seen in the two ε3/ε3 patients

*APOE*: apolipoprotein E gene; CI: confidence interval; CT: computed tomography; LOC: loss of consciousness; MMSE: Mini Mental State Exam; OEF: Operation Enduring Freedom; OIF: Operation Iraqi Freedom; TBI: traumatic brain injury.
